# Antibiotic Consumption 2017–2022 in 30 Private Hospitals in France: Impact of Antimicrobial Stewardship Tools and COVID-19 Pandemic

**DOI:** 10.3390/antibiotics13020180

**Published:** 2024-02-12

**Authors:** Pierre-Marie Roger, Diane Lesselingue, Anouk Gérard, Jacques Roghi, Pauline Quint, Sophie Un, Agnès Chincholle, Assi Assi, Odile Bouchard, Véronique Javaudin, Eric Denes

**Affiliations:** 1 Infectiologie, Clinique Les Fleurs, 332 ave F. Mistral, 83110 Ollioules, France; 2 Cellule Recherche et Enseignement, Groupe Elsan, Territoire Provence Alpes-Côte d’Azur, 83110 Ollioules, France; 3 Pharmacie, Clinique Jeanne d’Arc, 7 rue N. Saboly, 13200 Arles, France; 4 Pharmacie, Clinique Bouchard, 77 rue Dr Escat, 13006 Marseille, France; 5 Pharmacie, Clinique St Michel, Pl 4 Sept av Orient, 83100 Toulon, France; 6 Pharmacie, Clinique Inkermann, 84 Rte d’Aiffres, 79000 Niort, France; 7 Pharmacie, Hôpital Privé Océane, 11 rue Dr J. Audic, 56000 Vannes, France; 8 Pharmacie, Centre Médico-Chirurgical du Mans, 28 rue Guetteloup, 72100 Le Mans, France; 9 Infectiologie, Clinique Rhône Durance, 1750 ch Lavarin, 84000 Avignon, France; 10 Hygiène, Centre Chirurgical Montagard, 23 bd Gambetta, 84000 Avignon, France; 11 Infectiologie, Polyclinique de Limoges, Site Chénieux, 18 rue du Général Catroux, 87000 Limoges, France

**Keywords:** antibiotic consumption, defined daily doses, antimicrobial stewardship, tools, COVID-19

## Abstract

Our aim was to determine the impact of antimicrobial stewardship tools (ASTs) and the COVID-19 pandemic on antibiotic consumption (AC). We used the national software Consores^®^ to determine AC in DDD/1000 days of hospitalization from 2017 to 2022 in voluntary private hospitals in France. The ASTs considered were: 1. internal guidelines; 2. the list of antibiotics with restricted access; 3. the presence of an antibiotic referent or 4. an ID specialist; and 5. proof of an annual meeting on antimicrobial resistance. Institutions with dedicated units for COVID-19 patients were specified. In 30 institutions, the total AC varied from (means) 390 to 405 DDD/1000 DH from 2017 to 2022. Fluoroquinolones and amoxicillin/clavulanate consumption decreased from 50 to 36 (*p* = 0.003) and from 112 to 77 (*p* = 0.025), respectively, but consumption of piperacillin/tazobactam increased from 9 to 21 (*p* < 0.001). Over the study period, 10 institutions with ≤2 AST had lower AC compared to 20 institutions with ≥3 AST (*p* < 0.01). COVID-19 units opened in 10 institutions were associated with a trend toward higher macrolide consumption from 15 to 25 from 2017 to 2020 (*p* = 0.065) and with an acceleration of piperacillin/tazobactam consumption from 2020 to 2022 (*p* ≤ 0.003). Antibiotic consumption in 30 private hospitals in France was inversely related to the number of AST. The COVID-19 pandemic was associated with limited impact on AC, but special attention should be paid to piperacillin/tazobactam consumption.

## 1. Introduction

Antibiotic stewardship (AMS) is assigned to good antibiotic use, i.e., a lower antibiotic consumption (AC), to fight antimicrobial resistance and improve care [1]. The program for AMS includes several tools, among which organization, process and outcome measures must be in place in the present decade [2]. However, previous studies showed that the benefit of each tool is difficult to identify, even if specific means have been reported as being more efficient than others [3,4]. For example, audits and feedback are the most efficient way to improve the quality of antibiotic prescriptions, followed by pre-prescription authorization [5].

The core elements of the AMS program have been put in place in many countries and are associated with both a reduction of AC and better care [2,3,6,7]. Accordingly, AC was in a decreasing phase these last years in France, as illustrated by successive European reports on antimicrobial consumptions: expressed as Defined Daily Dose (DDD) per 1000 inhabitants per day, the total consumption of antimicrobials for systemic use was 25.7 in 2012 and 21.5 in 2021 [8].

Undoubtedly, the main challenge of the AMS policies is to ensure that their benefits persist over time. Because clinical practices vary according to new health technologies, the renewal of medical teams, and economic and resources constraints, it is possible to consider a progressive loss of efficiency of AMS tools. This is why audits of the appropriateness of antimicrobial use should be conducted regularly [9]. Moreover, a major difficulty in the health care system, such as the COVID-19 pandemic, can alter the performance of the AMS tools already in place. Previous reports showed that this pandemic was associated with an increase in the consumption of some drugs, such as azithromycin or doxycycline, but also in broad-spectrum antibiotics, notably during the first waves of the pandemic [10,11]. Because AC is subjected to both internal and external factors related to hospitals, we aimed to determine the current relationship between AC and both AMS tools and the COVID-19 pandemic.

## 2. Results

### 2.1. Main Characteristics of Institutions

Records of AC via Consores^®^ were provided by the institutions between April and June 2023, and involved 30 hospitals. The latter had medical and surgical activities in 23 cases (77%), surgical activities in only 6 cases (20%), and 1 institution only had medical activity. Fifteen institutions (47%) had an emergency department, and thirteen (43%) had an intensive care unit. The median [range] number of beds was 156 [45–300].

### 2.2. Antimicrobial Stewardship Tools

Regarding AMS tools already implemented in 2017, the most frequent one was internal guidelines available in 24 institutions (80%), followed by restricted access to some antibiotics (i.e., those with a significant ecological impact (e.g., carbapenem), those with a non-negligible financial cost and those that we feel should be protected (e.g., fluoroquinolones)) available in 15 institutions (50%), and the presence of an antibiotic referent and/or of an infectious disease (ID) specialist and/or an annual meeting on antimicrobial resistance, all of which were reported in 13 institutions (43%). An antibiotic referent is a medical doctor or a pharmacist or any other physician who is the resource for antibiotics questions. In total, 10 institutions (33%) had ≤2 AMS tools, and 20 institutions (67%) had ≥3 AMS tools.

### 2.3. Antimicrobial Consumption

**Figure 1:**
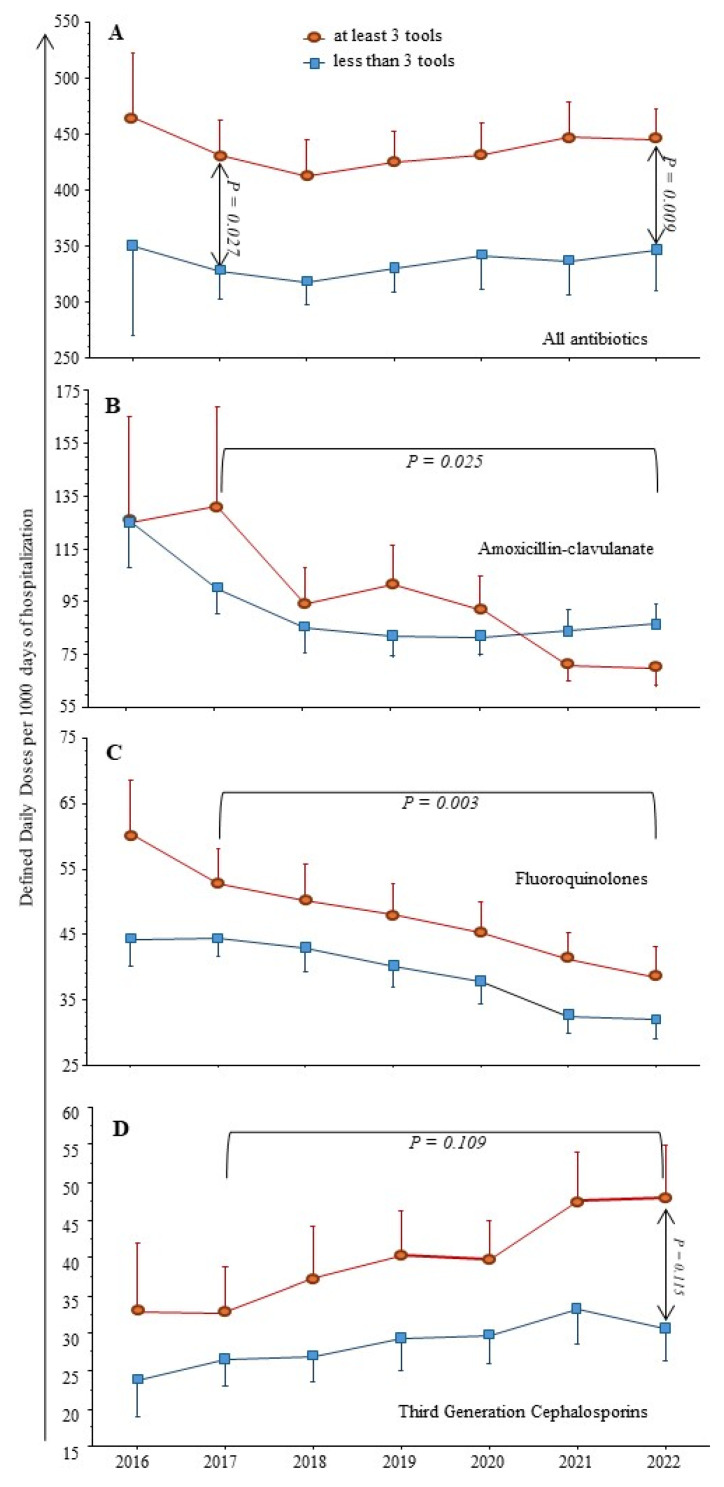
**Antibiotic consumption (AC) from 30 institutions from 2017 to 2022.** As the reports from Consores^®^ contain comparisons from previous year, data on AC in 2016 were available for 12 institutions. (**A**) Total AC was stable from 2017 to 2022 and significantly higher in institutions with ≥3 antimicrobial stewardship tools (*n* = 20, 67%). (**B–D**) In the same period, third-generation cephalosporins (cefotaxime, ceftriaxone, ceftazidime) consumption increased, while that of fluoroquinolones (ciprofloxacin, ofloxacin, levofloxacin) and amoxicillin/clavulanate decreased. Institutions with ≥3 AMS tools always had significantly higher AC compared to hospitals with ≤2 AMS tools.

As shown in Figure 1, from 2017 to 2022 the total AC was stable (means): from 390 to 405 DDD/1000 DH, but with a significant decrease of fluoroquinolones (FQ) and amoxicillin/clavulanate (AMC) from 50 to 36 (*p* = 0.003) and 112 to 77 (*p* = 0.025), respectively. In contrast, we observed a trend toward increased Third Generation Cephalosporins (TGC) consumption during the same period (*p* = 0.109) and a significant increase of piperacillin/tazobactam (Pip/taz) use over the study period from 9 to 21 (*p* < 0.001).

When comparing institutions with ≥3 AMS tools already in place at the beginning of the study period to other hospitals with ≤2 AMS tools, the latter always had significantly lower AC, regardless of the drug considered (see Figure 1).

**Figure 2:**
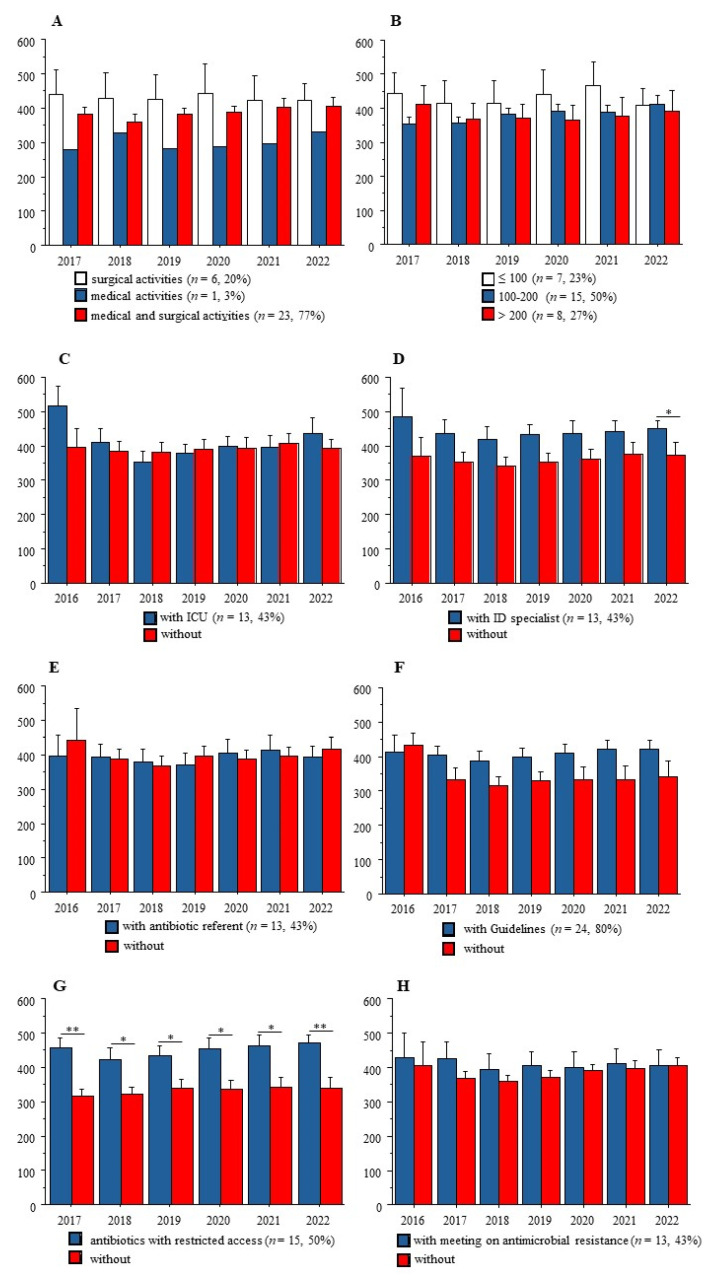
**Antibiotic consumption (AC) and main characteristics of the participating institutions.** (**A**) Medical and/or surgical activities; (**B**) number of beds in 3 groups; (**C**) presence of an Intensive Care Unit; (**D**) presence of an infection disease (ID) specialist; (**E**) presence of an antibiotic referent; (**F**) internal guidelines; (**G**) list of antibiotics with restricted access; (**H**) annual meeting on antimicrobial resistance and antibiotic consumption between AMS team and physicians. Only significant differences are indicated. *: *p* < 0.050; **: *p* < 0.010.

Figure 2 shows that at any time-point from 2017 to 2022, AC was not related to the medical and/or surgical activities of these institutions, nor to the number of beds. Also, among the five AMS tools, the list of antibiotics with restricted access was associated with significantly higher AC at the institutions’ level.

### 2.4. COVID-19 Pandemic and Antimicrobial Consumption

The COVID-19 pandemic might lead to an increase of antibiotic prescriptions for respiratory infections, i.e., AMC or ceftriaxone and/or macrolides, or even Pip/taz in the case of healthcare-associated pneumonia. Ten institutions (33%) had a dedicated unit for COVID-19 patients, and seven (23%) had a dedicated intensive care unit.

**Figure 3:**
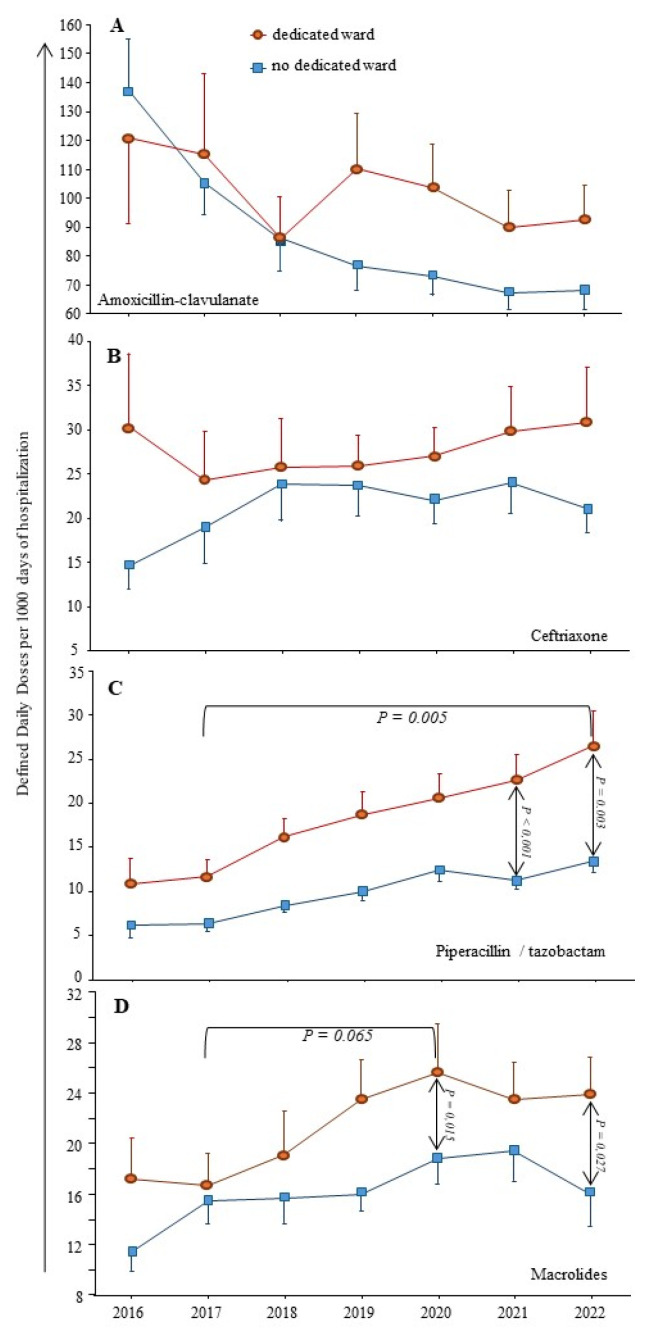
**Impact of the COVID-19 pandemic on antimicrobial consumption.** Dedicated wards for COVID-19 patients were opened in ten institutions. The consumption of most antibiotics used for respiratory infections is shown. (**A**) amoxicillin/clavulanate; (**B**) ceftriaxone; (**C**) piperacillin/tazobactam; (**D**) macrolides (including erythromycin; azithromycin; spiramycin; roxithromycin).

Figure 3 shows that most antibiotics prescribed for respiratory infections (AMC or ceftriaxone) were not used more often in institutions with dedicated units for COVID-19 patients when compared to others. Regarding macrolides, a trend toward a higher consumption was observed from 2017 to 2020, from 15 to 25 DDD/1000 DH, *p* = 0.065. Of note, azithromycin emerged in the top ten molecules during the COVID-19 waves in two institutions with dedicated units.

Also, the constant augmentation of Pip/taz use over the study period was significantly accelerated from 2020 to 2022 in institutions with dedicated units (*p* ≤ 0.003).

Lastly, the consumption of other antibiotic compounds, such as amoxicillin, carbapenems and aminoglycosides, did not vary significantly from 2017 to 2022, regardless of the comparison: number of AMS tools or COVID-19 pandemic.

## 3. Discussion

Our study shows that over 6 years, AC in participating hospitals was inversely correlated to the number of tools implemented for antimicrobial stewardship policy. Also, care related to COVID-19 patients was associated with an increase in macrolide use. Finally, the use of piperacillin/tazobactam increased significantly from 2017 to 2022 without a relationship with AMS tools, but with an accelerated consumption during COVID-19 waves.

It is worth remembering that private hospitals in France provide health care for at least 30% of the population, following AMS national recommendations; they participate in both medical education and clinical research.

Our study has several limitations. First, 30 out of 120 institutions among the same group gave their data, and we do not know if our sample was representative. Second, we do not know all significant changes at work in these institutions during the study period. For example, one institution had to close its emergency ward, and another had to close a medical ward, due to the lack of human resources in both cases. Also, we had observed a high turn-over of physicians in one hospital, leading to a brutal increase of AC (+90% in one year) [4]. Additionally, physicians’ reinforcements in overcrowded hospitals during the COVID-19 pandemic have been associated with inadequate knowledge of internal guidelines and heterogeneous practices [12]. Considering these limitations, we did not perform multivariate analyses.

In the current literature, AC is provided by the national healthcare system in France and/or surveillance reports from European or American institutes [8]. Few reports indicated AC at a lower scale, i.e., several institutions, trying to link AC to AMS tools. One French online survey described the AMS tools in 97 hospitals in 2020, showing that their implementations were common (between 84 to 95% of the participating institutions), but there were no data on AC, preventing any assessment of its effectiveness [2].

Our first result showing higher AC in institutions with ≥3 AMS tools is counterintuitive. It might suggest that these institutions with high AC knew their need for the implementation of most AMS tools, but the latter were still insufficient to fight antibiotic misuse. A large study performed in 2007 in 977 acute French hospitals showed a relationship between information technology support for prescriptions and lower antibiotic consumption [13]. Of note, there was no difference between public and private hospitals. However, in our work, all institutions used the same system of electronic patient records for several years, allowing a permanent link between the pharmacy, the laboratory and wards. Thus, the benefits of such an established technology might decrease over time. In a more recent study performed in 2013, an “intensity score” of antimicrobial stewardship was assessed in 44 academic centers in the US, but only the strategy component of the score was partly related to the amount of antimicrobials used [14]. Of note, ten years ago, we showed the absence of a relationship between AMS tools and the quality of bedside antibiotic treatments in four public hospitals in France [3].

Another counterintuitive result from our study is that the presence of an ID specialist in an institution was not associated with lower AC. This result could at least be explained by the paucity of the salary support to the AMS team: in the French survey cited above, most members received no salary support for their time spent on AMS activities, implying that ID specialists must develop their own activities [2]. Moreover, the antibiotic referents, who were appointed to this position by the administrator, always had other functions in the institution (pharmacist or any other medical function), limiting time spent on AMS policy. Accordingly, we observed that antibiotics with restricted access, always under the supervision of pharmacists, were associated with significantly higher AC (see Figure 2), suggesting a lack of human resources and/or an insufficient amount of time allocated to this AMS tool in practice, as reported previously [15]. Together, these data suggest the need for new tools in modern hospitals with high AC, because in the latter most rules relating to antimicrobial stewardship policy are already in place.

Our second result is that the COVID-19 pandemic was associated with few changes in AC. The measurable impact of the pandemic was a brief increase in macrolide consumption in 2020 compared to 2017. This result is in accordance with the putative efficacy of azithromycin against SARS-CoV-2, used during the first phase of the pandemic and finally infirmed in 2021 [16]. The weak impact of the pandemic on global AC has been reported, but always with some specific changes among beta-lactams or FQ. As an example, in Northern Ireland, the consumption of TCG as well as levofloxacin increased in the hospital setting [17]. We observed a significant increase in Pip/taz consumption each year from 2017 to 2022 (see Figure 3). Our result is in accordance with the latest report on the 2022-point prevalence survey on healthcare-associated infections and antimicrobial use in French healthcare facilities, showing that Pip/taz was the third drug to be prescribed, behind AMC and ceftriaxone: its prevalence was increased from 0.99% in 2017 to 1.64% in 2022 [18]. The accelerated Pip/taz consumption during the COVID-19 waves could be related to the increased rate of healthcare-associated infections in overcrowded intensive care units [19]. However, in clinical practices, we also observed that Pip/taz could replace AMC in community-acquired infections. This result suggested the introduction of specific measures to control its use.

Lastly, considering that AC is linked to antimicrobial resistance, one may ask about its potential increase during the COVID-19 pandemic. A meta-analysis including 28 studies, mainly from the United States, Italy and Brazil, reported that antimicrobial resistance did not increase significantly during the pandemic [20]. Moreover, infection prevention, control measures and the antimicrobial stewardship program were not significantly associated with rates of antimicrobial resistance [20].

## 4. Materials and Methods

### 4.1. Antibiotic Consumption

We used the national software Consores^®^ to determine the AC in Defined Daily Doses (DDD)/1000 days of hospitalization (DH) from 2017 to 2022 in voluntary-based private institutions working in a network in France [21]. Consores^®^ is a web tool recommended since 2015 by the French health authorities that allows for the analysis of antibiotic consumption in every hospital ward of a healthcare institution. The balance sheets of AC produced by the software were available in the first quarter of each year and included data from the previous year for comparison.

We systematically extracted the total amount of AC and more specifically the following drugs: amoxicillin, AMC, Pip/taz, carbapenems, ceftriaxone and total consumptions of TGC, FQ, and, in detail, ciprofloxacin, ofloxacin, levofloxacin, macrolides and aminoglycosides.

### 4.2. Antimicrobial Stewardship

Core elements of the AMS program have been published and are now largely all over the world in the hospital setting [1,21]. For our study, pharmacists and/or ID specialists from the participating institutions described the main AMS tools in place at the beginning of the study’s period: 1. existence of internal guidelines; 2. list of antibiotics with restricted access (i.e., those with a significant ecological impact (e.g., carbapenem), those with a non-negligible financial cost and those that we feel should be protected (e.g., FQ)) requiring a pre-prescription authorization from pharmacists; the restricted antibiotic list is dependent on the institutions and their own overprescribed molecules; 3. presence of an antibiotic referent (i.e., a pharmacist or microbiologist, or any other physician amenable to participating in antimicrobial stewardship) and/or 4. access to an ID specialist’s advice; and 5. proof of a bi-annual meeting between the AMS team and the physicians to discuss antimicrobial resistance and antibiotic consumption.

In accordance with our second goal, the institutions that had dedicated medical and/or intensive care units for COVID-19 patients were specified. Of note, four successive major waves were observed between March 2020 and October 2022 in mainland France [11].

### 4.3. Statistical Analysis

The data were analyzed with StatView software version 5.0, and statistical significance was established at α = 0.05. Continuous variables were compared with the Student *t*-test, the Mann–Whitney non-parametric test or the Kruskal–Wallis test when appropriate. Proportions were compared with the χ2 or Fisher exact test when appropriate. The ratio of antibiotic consumption from one year to another was calculated as follows: (Qx − Qy)/Qy. In all figures, the results are presented as means ± standard errors. Only significant *p* values or trends (*p* < 0.2) are shown; *p* values in horizontal brackets indicate comparisons over a period, while vertical arrows indicate comparisons between groups at a precise time-point.

## 5. Conclusions

The current AMS tools were partially inefficient in curbing antimicrobial consumption at hospitals’ level, despite the limited impact of the COVID-19 pandemic on antibiotic use. In order to obtain better results in the mid-terms, antimicrobial stewardship policies would need to be renewed. 

## Data Availability

The data used during the current study are available from the corresponding author on reasonable request.

## References

[1] Core Elements of Hospital Antibiotic Stewardship Programs: Antibiotic Use|CDC. https://www.cdc.gov/antibiotic-use/core-elements/hospital.html.

[2] Binda F., Tebano G., Kallen M.C., Ten Oever J., Hulscher M.E., Schouten J.A., Pulcini C. (2020). Nationwide survey of hospital antibiotic stewardship programs in France. Med. Mal. Infect..

[3] Etienne P., Roger P.-M., Brofferio P., Labate C., Blanc V., Tiger F., Négrin N., Léotard S. (2011). Antimicrobial stewardship program and quality of antibiotic prescriptions. Med. Mal. Infect..

[4] Roger P.-M., Espinet A., Ravily D., Meyer M.-J., Moll F., Montera E., Rancezot A., Dautezac V., Pantaloni O. (2022). Simplified therapeutic guidelines: The main tool of antimicrobial stewardship programs associated with optimal antibiotic therapy. Eur. J. Clin. Microbiol. Infect. Dis..

[5] Tamma P.D., Avdic E., Keenan J.F., Zhao Y., Anand G., Cooper J., Dezube R., Hsu S., Cosgrove S.E. (2017). What is the more effective antibiotic stewardship intervention: Preprescription authorization or postprescription review with feedback?. Clin. Infect. Dis..

[6] Schuts E.C., Hulscher M.E.J.L., Mouton J.W., Verduin C.M., Stuart J.W.T.C., Overdiek H.W.P.M., van der Linden P.D., Natsch S., Hertogh C.M.P.M., Wolfs T.F.W. (2016). Current evidence on hospital antimicrobial stewardship objectives: A systematic review and meta-analysis. Lancet Infect. Dis..

[7] Zhou J., Ma X. (2019). A survey on antimicrobial stewardship in 116 tertiary hospitals in China. Clin. Microbiol. Infect..

[8] Antimicrobial Consumption in the EU/EEA (ESAC-Net)—Annual Epidemiological Report for 2021. https://www.ecdc.europa.eu/en/publications-data/surveillance-antimicrobial-consumption-europe-2021.

[9] Mendelson M., Morris A.M., Thursky K., Pulcini C. (2020). How to start an antimicrobial stewardship programme in a hospital. Clin. Microbiol. Infect..

[10] Friedli O., Gasser M., Cusini A., Fulchini R., Vuichard-Gysin D., Tobler R.H., Wassilew N., Plüss-Suard C., Kronenberg A. (2022). Impact of the COVID-19 pandemic on inpatient antibiotic consumption in Switzerland. Antibiotics.

[11] Khan S., Hasan S.S., Bond S.E., Conway B.R., Aldeyab M.A. (2022). Antimicrobial consumption in patients with COVID-19: A systematic review and meta-analysis. Expert Rev. Anti-infective Ther..

[12] Viel S., Markowicz S., Ait-Medjber L., Ouissa R., Delta D., Portecop P., Foucan T., Roger P.-M. (2022). Dedicated team to ambulatory care for patients with COVID-19 requiring oxygen: Low rate of hospital readmission. Int. J. Infect. Dis..

[13] Amadeo B., Dumartin C., Parneix P., Fourrier-Réglat A., Rogues A.-M. (2011). Relationship between antibiotic consumption and antibiotic policy: An adjusted analysis in the French healthcare system. J. Antimicrob. Chemother..

[14] Pakyz A.L., Moczygemba L.R., Wang H., Stevens M.P., Edmond M.B. (2015). An evaluation of the association between an antimicrobial stewardship score and antimicrobial usage. J. Antimicrob. Chemother..

[15] Kamel A.M., Monem M.S.A., Sharaf N.A., Magdy N., Farid S.F. (2022). Efficacy and safety of azithromycin in Covid-19 patients: A systematic review and meta-analysis of randomized clinical trials. Rev. Med. Virol..

[16] Aldeyab M.A., Crowe W., Karasneh R.A., Patterson L., Sartaj M., Ewing J., Lattyak W.J., Al-Azzam S., Araydah M., Elhajji F.D. (2023). The impact of the COVID-19 pandemic on antibiotic consumption and prevalence of pathogens in primary and secondary healthcare settings in Northern Ireland. Br. J. Clin. Pharmacol..

[17] Enquete Prevalence IAS 2022.pdf. https://www.cpias-ile-de-france.fr/surveillance/enp/2022/SpF-ENP-2022-Guide-Enqueteur.pdf.

[18] Abubakar U., Awaisu A., Khan A.H., Alam K. (2023). Impact of COVID-19 pandemic on healthcare-associated infections: A systematic review and meta-analysis. Antibiotics.

[19] Langford B.J., Soucy J.-P.R., Leung V., So M., Kwan A.T., Portnoff J.S., Bertagnolio S., Raybardhan S., MacFadden D.R., Daneman N. (2023). Antibiotic resistance associated with the COVID-19 pandemic: A systematic review and meta-analysis. Clin. Microbiol. Infect..

[20] Boussat S., Demoré B., Lozniewski A., Aissa N., Rabaud C. (2012). How to improve the collection and analysis of hospital antibiotic consumption: Preliminary results of the ConsoRes software experimental implementation. Med. Mal. Infect..

[21] Santé Publique France Enquêtes Flash: Évaluation de la Circulation des Variants du SARS-CoV-2 en France. Saint-Maurice: Santé publique France. https://www.santepubliquefrance.fr/etudes-et-enquetes/enquetes-flash-evaluation-de-la-circulation-des-variants-du-sars-cov-2-en-france#block-33727.

